# Animal Health Service Delivery in Crop-Livestock and Pastoral Systems in Ethiopia

**DOI:** 10.3389/fvets.2021.601878

**Published:** 2021-06-07

**Authors:** Solomon Gizaw, Mengistu Woldehanna, Habtamu Anteneh, Gewado Ayledo, Fasil Awol, Gebreegziabher Gebreyohannes, Berhanu Gebremedhin, Barbara Wieland

**Affiliations:** ^1^International Livestock Research Institute (ILRI), Addis Ababa, Ethiopia; ^2^Ministry of Agriculture, Addis Ababa, Ethiopia; ^3^Ethiopian Veterinary Association (EVA), Addis Ababa, Ethiopia

**Keywords:** gender, systems, PCA, Ethiopia, health services

## Abstract

Livestock diseases are a priority problem for livestock keepers throughout Ethiopia. Livestock keepers have also singled out poor animal health service delivery, which is largely the domain of the public sector, as the major constraint to improving animal health and productivity. In the current study, we describe the animal health service delivery system and compile from five questionnaire surveys involving 4,162 livestock keepers to characterize animal health service delivery in Ethiopia. The mapping of the animal health service delivery system along the livestock value chain clearly highlights the role of informal animal health services and variations of roles of the private sector. Also, the survey results clearly showed that livestock keepers' access to, use of and satisfaction with animal health services significantly varied across livestock production systems, geographic locations, socioeconomic strata, and service providers. Livestock keepers in crop-livestock and agropastoral systems had 5.5 (odds ratio = 5.453, *P* = 0.000) and 2.5 (odds ratio = 2.482, *P* = 0.000) times more access to services in reference to the pastoral system. In reference to private veterinary clinics, livestock keepers reported higher access to services provided by all the other service providers, particularly to services provided by extension agents, drug shops and CAHWs. Similarly, better access was reported by male than female (odds ratio = 1.098; *P* = 0.025) and wealthier than poorer (odds ratios = 1.40–1.79; *P* = 0.000) farmers and pastoralists. In general, low access to services was reported, 32.7, 25.2, and 19.3% of the respondents reporting access in crop-livestock, agropastoral and pastoral systems, respectively. Effective demand for services was evaluated through proxy variables, namely number of visits to service providers and health expenditures over a year. Highland farmers used the services more often than pastoralists (odds ratio = 2.86; *P* = 0.000), but pastoralists' expenses were significantly higher. Wealth (measured by livestock owned), gender and age also had significant effects on the use of services and expenditure on services. Satisfaction with services was evaluated based on four measures, namely availability (av), accessibility (ac), quality (qw), and timeliness (tm) of services. The average scores (out of 10) for av, ac, qw, and tm were 6.1, 5.9, 6.2, and 5.7, respectively. Principal component analysis was conducted to derive the latent variable “satisfaction” from the four measures, extracted only one factor, indicating the four variables are measuring the same construct (satisfaction). Regressing the latent variable satisfaction on the four measures gave significant (*P* = 0.000) *b* values of 0.22, 0.20, 0.13, and 0.14 for av, ac, qw, and tm, respectively, indicating strong relationships between the latent variable satisfaction and its measures. There was a significant dissatisfaction with the public sector, with average scores of 0.06 and 0.19 for the public and private service providers, respectively. It can be concluded that livestock keepers in remote regions of the country, pastoralists, women, poorer, and older livestock keepers have less access to services. Satisfaction with services is low to medium and the major concerns of livestock keepers appears to be availability and accessibility of services. Based on our findings, we recommend an integrated, multi-sectoral involvement to improve the veterinary service delivery through improved veterinary infrastructure, public-private partnership, and animal health information system across the various livestock production systems.

## Introduction

Ethiopia is endowed with huge livestock resources comprising of 61.5 million heads of cattle, 33.0 million sheep, 39.0 goats, 59.4 million poultry, 11.96 million equines, 1.76 million camels and 7.1 million beehives. A production of 3.3 billion liters of cow milk, 282.2 million liters of camel milk, 151.47 million eggs and 58.6 million kg honey is being recorded per annum (1).

The livestock sub-sector in Ethiopia plays vital roles in ensuring food security, provision of traction power, generation of rural income and employment at the household level as well as national economic growth through foreign exchange earnings but is also culturally important. However, the contribution of this resource to the national economy is not commensurate to the huge national potential. This mismatch is mostly caused by the widespread prevalence of many infectious and parasitic diseases (2–4) which drastically reduce the production and productivity of livestock through morbidity, mortality and market restrictions (5, 6).

Veterinary services are defined as all the public and private players that implement animal health, welfare measures and other standards and recommendations to ensure effectiveness of the system, under the control of the Veterinary Authority (7). It implies that, strong, transparent and credible veterinary services provided by both, the public and private sector, are necessary for enhancing the performance of animal health systems by mitigating animal disease risks, ensuring sustainable economic development of vulnerable producers, and limiting the public health risks posed by zoonotic diseases. Strong veterinary services also provide confidence for private sector investment from both individual farmers and livestock enterprises across the livestock value chains.

Despite various reform efforts over the last decades, provision of adequate veterinary services to smallholder farmers has remained a serious challenge in Ethiopia. Particularly, the coverage and quality of veterinary services are less than satisfactory across the different livestock production systems (2, 8, 9). On top of this, despite few pilot studies, mostly done for academic purposes, in specific areas of the central highlands, there is lack of comprehensive, well-documented and reliable information regarding the core determinants of animal health services delivery in reference to the livestock production systems across different bio-geographic and socio-economic conditions of Ethiopia. Therefore, a review of stakeholders involved in the provision of animal health related services and a household survey was conducted in 9 regional national states to bridge this gap.

## Materials and Methods

### Stakeholder Analysis

A descriptive analysis of the animal health service value chains in Ethiopia was conducted to provide the context to the detailed quantitative household surveys (Section Household Survey). The analysis was built on comprehensive surveys of actors involved in animal health service provision in eight of the nine administrative regions in Ethiopia (see details in Table 1) as well as review of the literature. The literatures consulted included a survey of health service delivery in four administrative regions (10), the Veterinary Services Rationalization Roadmap for Ethiopia (11) and The Livestock Master Plan for Ethiopia (5).

**Table 1:** Source of data and data structure.

**Region**	**Production system^a^**	**No. of woredas sampled^b^**	**No. of Households sampled**	**Project^c^**
Afar	Pastoral	6	191	DRSLP I
	Pastoral/AP	7	294	RPLRP
Amhara	Mixed	4	216	HEARD
	Mixed	11	360	LFSDP
Benishangul-Gumz	Mixed	3	90	LFSDP
Gambela	Mixed	1	60	LFSDP
Oromia	Pastoral	12	540	DRSLP II
	Mixed/P	4	241	HEARD
	Mixed/P	16	479	LFSDP
	Pastoral/AP	9	378	RPLRP
SNNP	Pastoral	11	330	DRSLP II
	Mixed/P	9	270	LFSDP
	Pastoral/AP	6	252	RPLRP
Somali	Pastoral/AP	8	371	RPLRP
Tigray	Mixed	4	120	LFSDP
Overall		111	4,162	

a *AP, agropastoral; Mixed, mixed crop-livestock system*.

b *Four and two kebeles were sampled per woreda for the DRSLP/RPLRP/LFSDP and for the HEARD projects, respectively*.

c *DRSLP, Drought Resilience of Sustainable Livelihood program; RPLRP, Rural Pastoral Livelihood and resilience Project; LFSDP, Livestock and Fishery Sector development Project; HEARD, Health of Ethiopian Animals for Rural Development*.

### Household Survey

#### Source of Data

The data for this study were obtained from baseline studies, conducted to collect data for the purpose of evaluating project impacts at the end of the projects, of five livestock development projects in Ethiopia: Drought Resilience of Sustainable Livelihood program (DRSLP I and II), Regional Pastoral Livelihoods Resilience Project (RPLRP), Livestock and Fishery Sector Development Project (LFSDP) and Health of Ethiopian Animals for Rural Development (HEARD) (Table 1). The DRSLP, RPLRP (12) and LFSDP projects are implemented by the Ministry of Agriculture (MoA). The DRSLP and RPLRP projects aim to improve the resilience capacity and livelihoods of the pastoral and agro-pastoral communities in Ethiopian Somali, Afar, Oromia, and the Southern Nations, Nationalities and Peoples region (SNNP). The two projects are funded by African Development Bank and World Bank, respectively. The LFSDP project, initiated in 2019, is funded by the World Bank to strengthen the livestock and fishery development and operates in the mixed crop-livestock system in the highland and mid-highland areas. The HEARD project is led by the MoA, jointly implemented by the International Livestock Research Institute (ILRI), the Ethiopian veterinary association (EVA), and three regional states (Somali, Oromia and Amhara regions). The project is initiated in 2019 and supported by the European Union. All the five projects are still ongoing, and their impacts have not yet been evaluated.

#### Sampling and Data Collection

Both purposive and stratified clustered sampling approaches were used to draw representative samples for the household surveys for the projects described above. Sampling was stratified at different stages, namely by livelihood zones (pastoral, agropastoral and mixed crop-livestock systems) and administrative zones at the levels of regional states, woredas and Kebeles (the smallest administrative unit). Regions were selected purposively as per the projects aims and design. The projects covered eight of the nine regions in Ethiopia. Woredas within regions and kebeles within woredas were selected randomly considering the livelihood zones. Households were selected randomly considering gender, age and livestock holdings. The sampling frame and data structure is shown in Table 1.

The livelihood systems overlapped with agro-ecological zones, the pastoral/agro-pastoral systems and the crop-livestock zones being located mainly in the lowlands (mostly below 500 meters above sea level) and highlands (commonly above 2,000 m a.s.l), respectively, though mixed crop-livestock production is also found in lower altitudes between 1,000 and 2,000 m a.s.l. The DRSLP and RPLRP projects operated in the arid and semi-arid lowlands, whereas the LFSDP and HEARD projects operate in the highland (above 2,000 m a.s.l), midland and lowland areas (1,000 and 2,000 m a.s.l).

The data were collected using household surveys with structured questionnaires. All the data were collected by ILRI as a baseline for the five projects. The data were collected on various aspects of the households including household demographics, physical assets, livestock holding and composition, crop technology adoption and use, sources of livelihoods, and access to services. The data for the current analysis included household demographics, animal health services, which included the types of services and the service providers, access to services by the livestock keepers, frequency of visits to health service providers, expenditure on services, and satisfaction with the services.

#### Data Analysis

Both descriptive and inferential statistical analyses were used. For descriptive analyses, the response data were disaggregated by bio-geographic zones (production systems, geographic regions), socio-economic strata (gender, age, wealth status measured by livestock holdings), and the types of service providers. Proportions of the sampled households with the alternative responses (e.g., access or no access to services) within each bio-geographic zone and socio-economic strata were calculated to assess the availability and accessibility of the services and satisfaction of the livestock keepers with the services. Proportions were compared using chi-squared tests.

Access to services was coded as a binary variable (1 = access, 0 = no access). Access was defined to include the availability of a service in a location and its affordability for the various livestock keepers. It was hypothesized that access to services could be determined by bio-geographic and socio-economic circumstances and the types of service providers. A binary logistic regression was fitted to model the probability or likelihood of a livestock keeper under a certain bio-geographic and socio-economic condition accessing a service in reference to livestock keepers in a different bio-geographic and socio-economic condition.

Effective demand for services was evaluated through proxy variables, namely number of visits to service providers and health expenditures over a year. Differences in effective demand across the different bio-geographic and socio-economic conditions were analyzed using a generalized linear model procedure fitting a natural logarithmic transformation of the data to meet the normal distribution assumption for a linear model analysis and fitting a Poisson distribution for the count data of number of visits to service providers. In all analyses, comparisons between the likelihood of a livestock keeper under a certain socio-economic condition having more access to services or making more visits in reference to other conditions were made using odds ratios as suggested by Abeyasekera (13) and Agresti (14).

Satisfaction of livestock keepers with services provided by the different service providers was assessed based on respondents' scoring (out of 10) of four variables assumed to measure satisfaction, namely availability, quality, accessibility, and timeliness of services. Principal component analysis was conducted on the four variables to extract a single measure of satisfaction. Mean scores on the transformed variable were calculated for each bio-geographic and socio-economic categories to measure satisfaction of livestock keepers with the different service providers.

## Results

### Health Service Value Chains

The mapping of actors involved in the provision of animal health services (Table 2) showed that the value chain structure is influenced by the administrative organization of the government of Ethiopia. The federal ministry of agriculture and the regional livestock bureaus are the enabling bodies in their respective domains. Thus, federal and regional policies and strategies are the key enabling instruments for improving health services.

**Table 2:** Value chain analysis of animal health service delivery (actors and roles) in Ethiopia.

**Actors**	**Location**	**Sector**	**Education**	**Presence**	**Role (secondary role in parentheses)**	**Remark**
*Kebele* health posts/clinics (type D)	Villages	Public	Dipl., BSc, DVM	1 for 2-3 kebeles	Vaccination; clinical services	Growing: 1 in each *kebele* in some cases
	Villages	Private	Diploma; BSc; DVM	Very few	Clinical services	Mainly at district level
*Kebele* drug shops	Villages	Public	Diploma; BSc; DVM	1 for 2 *kebele*	Drug sale	Part of health post
	Villages	Private	Diploma; BSc; DVM	Few (>clinics)	Drug sale (clinical services)	Illegal clinical service
CAHWs	Villages	Private	Certificate	Very few	Vaccination (clinical services)	Pastoral areas
District clinics (type C)	Towns	Public	DVM; MVSc	Every district	Coordinate vaccination; clinical services	
	Towns	Private	Diploma; BSc; DVM	Few	Clinical services	
District drug shops	Towns	Public	DVM; MVSc	Every district	Drug sale	Part of clinic
	Towns	Private	Diploma; BSc; DVM	Few	Drug sale (clinical services)	Illegal clinical service
Large-scale pharmaceutical importers/distributors	Federal Capital	Private	DVM; MVSc	Few	Distribution to regional bureaus, private whole sealers, private shops/clinics	
Small/medium scale drug importers/distributors	Regional capitals	Private	DVM; MVSc	Few	Distribution to small clinics, drug shops	About 10-20
District laboratories	District towns	Public	DVM; MVSc	Few	Minor diagnosis	
Regional laboratories	Regional/zonal capitals	Public	MVSc; PhD	1-2 per region	investigation, surveillance, food safety, capacity building	Developing regions?
National Veterinary Institute		Public	MVSc; PhD	One	Vaccine production	High contribution
Federal laboratories (National Animal Health Diagnostic and Investigation Center)		Public	MVSc; PhD	1	Diagnostics; surveillance, food safety, capacity development	
VDFACA (Veterinary Drugs and Feed Accreditation and certification Authoriy)		Public	DVM; MVSc		Quality control	Ill-equipped; weak regional branch
Abattoirs	Regional/zonal capitals	Public	DVM; MVSc		Meat inspection	
Federal Livestock Ministry		Public	DVM; MVSc		Enablers; regulators, certification	
Regional Livestock Bureaus^a^		Public	DVM; MVSc	1/region	Enablers; regulators, certification	
Livestock keepers		Priavte	0-12 grade		Passive surveillance	Few graduates
Livestock extension agents	At all level	Public	Diploma, BSc, MSc		Livestock production advisory, coordinating/facilitating health services (esp. vaccination)	
Prof. associations, Univ., Research^b^	Federal, regional	Public	DVM; MVSc; PhD	Quite a few	Technical support	Minimal contribution

a *Zonal, district and kebele structures*.

b *Federal, regional, and international research institutes*.

Both the public and private sectors are involved in animal health service delivery. The private sector is mainly involved in drug sales, and that is mainly in district towns, while clinical or diagnostic services are very minimal and are available only in and around urban areas. It is estimated that private drug shops and clinics account for 75 and 25% of the private service centers, respectively. For instance, in a 2001 estimate, the number of private importers, clinics + drug shops, clinics only, animal health posts and drug shops were 127, 94, 40, 35, and 180, respectively (15).

**Figure 1:**
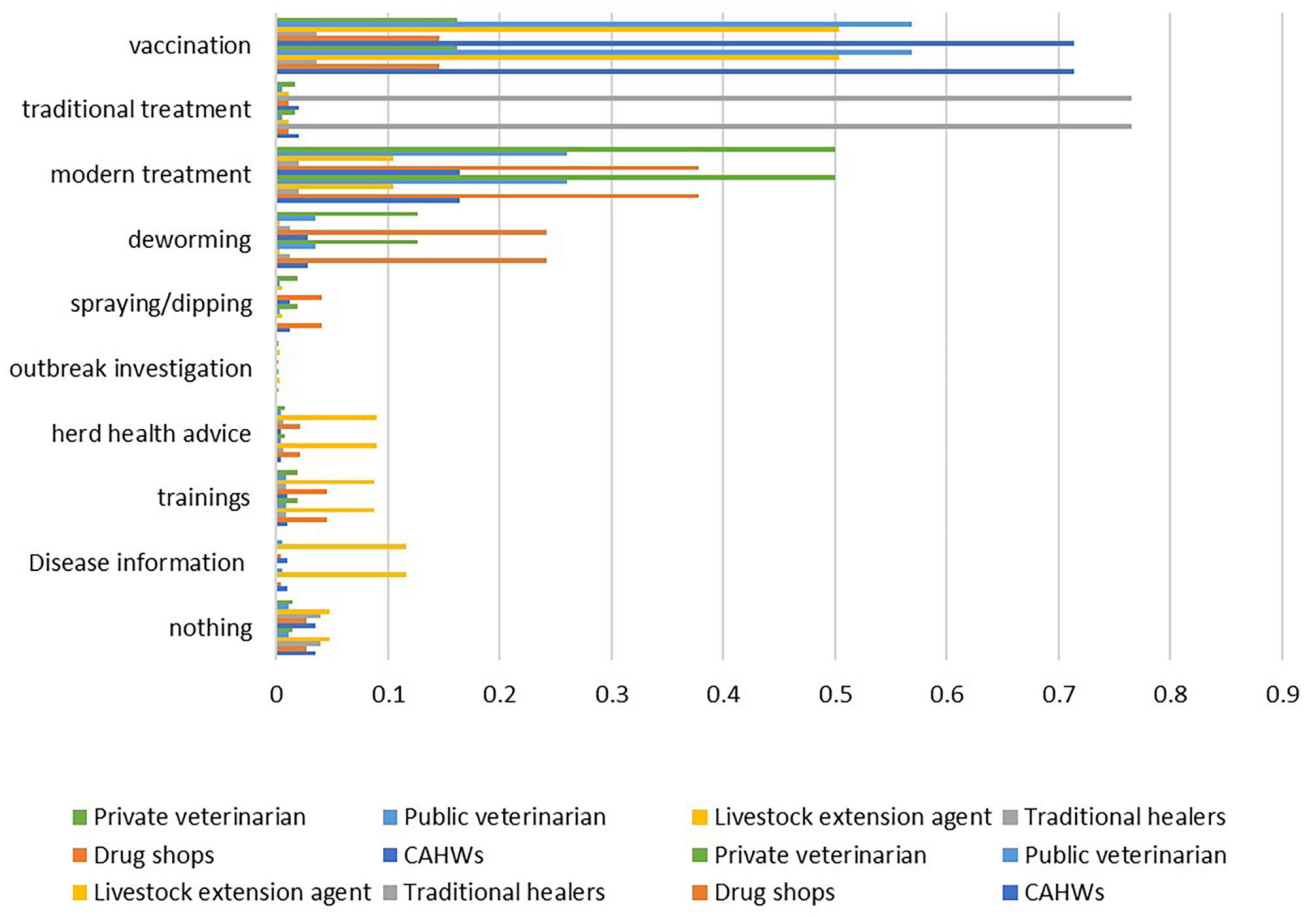
Percentage of respondents reporting animal health service providers and their primary services in Ethiopia (CAHWs, community-based animal health workers).

Yet, importation and distribution of pharmaceuticals, including to the public sector, is predominantly the private sector's domain, reflecting the fact that this is likely the main domain where profits are possible. Effective delivery of animal health services is hampered by absence or under-equipped and under-staffed district laboratories, inefficient delivery of supplies, severe shortage of transportation means to deliver services, and poor quality drugs/vaccines, unethical practices both by the public and private sector practitioners, and importantly absence of favorable enabling environment for the private sector. The key public and private sector service providers identified through household surveys included livestock extension agents, public/official veterinarians and CAHWs, drug shops, traditional healers, and private veterinarians (Figure 1). Vaccination is primarily provided by the CAHWs (71.4% of respondents), the public veterinary service (56.9%), and extension agents (50.4%, whose role would likely be limited to awareness creation and organizing the vaccination campaigns. The private veterinary clinics provided most of the clinical services compared to the public veterinarians, the percentage of respondents claiming to get services from the private and the public sectors being 50 and 26%, respectively. The drug shops also provided clinical services including most of the deworming services, violating the limitations of their professional business license, as reported by 37.8 and 24.2% of the respondents, respectively. Advice and trainings on herd health and information on diseases is virtually absent despite its potential importance in the traditional livestock production systems. These services were mainly provided by the extension agents.

### Animal Health Services

Animal health services provided in Ethiopia include vaccination, modern (clinical services by professional and paraprofessionals) and traditional treatments, GIT parasite (deworming) and external parasite (spraying/dipping) controls, disease outbreak investigations and information on diseases outbreaks, herd health advices, and trainings (Figure 1). The services most frequently reported by the livestock keepers were vaccination and modern treatments, being reported by 40.9 and 21.4% of the respondents, respectively. External parasite control, outbreak investigation, herd health advices, delivery of trainings and disease information are the least available services, reported by 0.2 to 4.0% of the respondents.

Services provided were similar across the three major livestock production systems in Ethiopia, though the proportion of respondents reporting the different services varied significantly across the systems (Table 3). Availability of vaccination and traditional treatment services were reported by a larger proportion of the respondents in pastoral and agropastoral systems (*P* < 0.05). Advice on herd health management, trainings and information on disease outbreaks were more available in mixed crop-livestock system (*P* < 0.05).

**Table 3:** Percentage of respondents reporting the primary health services provided within pastoral, agropastoral and mixed crop-livestock systems in Ethiopia.

	**Crop-livestock**	**Agropastoral**	**Pastoral**
Vaccination	33.8%a	50.1%b	43.2%c
Traditional treatment	5.8%a	11.1%b	12.2%b
Modern treatment^a^	22.6%a	21.1%a, b	19.5%b
Deworming	6.9%a	5.1%b	8.3%a
Spraying/dipping	1.2%a	1.1%a	1.6%a
Outbreak investigation	0.2%a	0.1%a	0.2%a
Herd health advice	5.4%a	1.2%b	1.7%b
Trainings	5.2%a	4.0%a	1.8%b
Disease information	5.5%a	1.4%b	3.0%c
Nothing	2.9%a	3.1%a	3.5%a
Other services	10.5%a	1.6%b	5.1%c

*Each subscript letter denotes a subset of system categories whose column proportions do not differ significantly from each other at the 0.05 level. Modern treatment refers to clinical services provided by professionals and paraprofessionals*.

### Access to Services

Multinomial logistic regression analyses showed that biogeographic, socioeconomic, and institutional factors determined access to animal health services (Table 4). Livestock keepers in crop-livestock and agropastoral systems had 5.5 (odds ratio = 5.453, *P* = 0.000) and 2.5 (odds ratio = 2.482, *P* = 0.000) times more access to services in reference to the pastoral system. Within production systems, administrative regions located in the central part of the country and/or with developed infrastructure had significantly more access to services than peripheral regions. For instance, livestock keepers in Amhara and Oromia regions located in the central part of the country in crop-livestock system and in Oromia region in agropastoral and pastoral systems reported better access to services than the reference regions which are located in the border area or have less developed infrastructures (Table 4).

**Table 4:** Biogeographic, socioeconomic, and institutional determinants of access to animal health services in Ethiopia.

**Parameter**	***B***	**SE**	**Sig (*P*)**	**Exp(B)^a^**
Intercept	−4.212	0.118	0.00	0.015
System = Crop-livestock	1.696	0.128	0.00	5.453
System = Agropastoral	0.909	0.186	0.00	2.482
System = Pastoral	0^b^	.	.	1
Region = Amhara (system = crop-livestock)	0.510	0.096	0.000	1.665
Region = Oromia (system = crop-livestock)	0.229	0.095	0.016	1.257
Region = SNNP (system = crop-livestock)	−0.205	0.108	0.057	0.815
Region = Tigray (system = crop-livestock)	0^b^	.	.	1
Region = Afar (system = Agropastoral)	−0.731	0.208	0.00	0.482
Region = Oromia (system = Agropastoral)	0.950	0.167	0.00	2.585
Region = SNNP (system = Agropastoral)	0.145	0.171	0.39	1.156
Region = Somali (system = Agropastoral)	−1.025	0.216	0.00	0.359
Region = Benishangul (system = Agropastoral)	0.747	0.189	0.00	2.11
Region = Gambella (system = Agropastoral)	0^b^	.	.	1
Region = Afar (system = Pastoral)	0.131	0.111	0.238	1.14
Region = Oromia (system = Pastoral)	2.004	0.097	0.00	7.418
Region = SNNP (system = Pastoral)	0.846	0.114	0.00	2.331
Region = Somali (system = Pastoral)	0^b^	.	.	1
Male HH head	0.094	0.042	0.025	1.098
Female HH head	0^b^	.	.	1
Herd size				
Medium (10.5)	0.425	0.044	0.00	1.53
Large (25.7)	0.542	0.049	0.00	1.72
Very large (99.8)	0.693	0.057	0.00	1.999
Small (2.8)	0^b^	.	.	1
HH age category (average)				
1st quartile (29.9)	−0.041	0.043	0.343	0.96
2nd quartile (39.4)	0.061	0.046	0.180	1.063
3rd quartile (47.0)	0.069	0.044	0.117	1.072
4th quartile (61.4)	0^b^	.	.	1
Service providers				
CAHWs	1.143	0.063	0.00	3.136
Drug shop	1.396	0.062	0.00	4.04
Traditional healer	0.492	0.067	0.00	1.636
Extension agent	2.077	0.061	0.00	7.978
Public vets	1.535	0.062	0.00	4.642
Private vets	0^b^	.	.	1

a *(Exp(B): the odds of reporting access to service in reference to no access)*.

b *Set to zero because this parameter is redundant as it is the reference category*.

Determinants of access to animal health services were similar across the three livestock systems studied (Table 5). Male, older and wealthier livestock keepers had a higher chance of access to animal health services. However, there was no gender difference in the pastoral system and medium-aged farmers had higher access to services than older in crop-livestock system.

**Table 5:** Biogeographic, socioeconomic, and institutional determinants of access to animal health services in mixed crop-livestock, agropastoral and pastoral livestock production systems in Ethiopia.

	**Crop-livestock system**				**Agropastoral system**				**Pastoral system**			
**Parameter**	***B***	**SE**	**Sig (*P*)**	**Exp(B)^b^**	***B***	**SE**	**Sig (*P*)**	**Exp(B)^b^**	***B***	**SE**	**Sig (*P*)**	**Exp(B)^b^**
Intercept	−2.009	0.0900	0.000	0.134	−3.366	0.1829	0.000	0.035	−3.225	0.1982	0.000	0.040
Male HH head	0.119	0.0566	0.036	1.126	0.259	0.1082	0.017	1.295	−0.030	0.0787	0.706	0.971
Female HH head	0^a^			1	0^a^			1	0^a^			1
Herd size											
Medium (10.5)	0.647	0.0562	0.000	1.910	0.299	0.0861	0.001	1.349	0.473	0.1544	0.002	1.604
Large (25.7)	0.982	0.0709	0.000	2.669	0.388	0.0788	0.000	1.474	0.408	0.1422	0.004	1.503
Very large (99.8)	1.054	0.1616	0.000	2.870	0.259	0.0853	0.002	1.296	0.365	0.1361	0.007	1.440
Small (2.8)	0^a^			1	0^a^			1	0^a^			1
HH head age category (average age)												
1st quartile (29.9)	0.063	0.0697	0.367	1.065	−0.209	0.0751	0.005	0.811	−0.197	0.0774	0.011	0.821
2nd quartile (39.4)	0.050	0.0703	0.475	1.052	−0.074	0.0849	0.383	0.929	−0.115	0.0847	0.173	0.891
3rd quartile (47.0)	0.194	0.0666	0.004	1.214	−0.249	0.0822	0.002	0.780	−0.030	0.0843	0.726	0.971
4th quartile (61.4)	0^a^			1	0^a^			1	0^a^			1
Service providers												
CAHWs	−0.678	0.1013	0.000	0.508	2.178	0.1447	0.000	8.833	2.577	0.1423	0.000	13.160
Drug shop	0.664	0.0835	0.000	1.943	2.233	0.1444	0.000	9.330	2.041	0.1444	0.000	7.696
Traditional healer	−0.401	0.0956	0.000	0.670	1.531	0.1496	0.000	4.625	1.276	0.1516	0.000	3.581
Extension agent	2.213	0.0842	0.000	9.140	2.443	0.1436	0.000	11.506	1.917	0.1452	0.000	6.798
Public vets	1.410	0.0816	0.000	4.095	2.182	0.1447	0.000	8.865	1.380	0.1502	0.000	3.975
Private vets	0^a^			1					0^a^			1

a *Set to zero because this parameter is redundant as it is the reference category*;

b *(Exp(B): the odds of reporting access to service in reference to no access)*.

In reference to private veterinary clinics, livestock keepers reported higher access to services provided by all the other service providers, particularly to services provided by extension agents, drug shops, and CAHWs. Similarly, male headed households and wealthier livestock keepers (measured by their herd sizes) had more access to services. The proportions of respondents in the different system, gender, and age categories reporting access to services are presented in Table 6.

**Table 6:** Proportion (%) of male and female respondents with different age groups and herd sizes in different production systems reporting access to animal health services by different service providers in eight regions of Ethiopia.

**Production systems**	**CAHWs**	**Drug shops**	**Traditional healers**	**Extension agent**	**Public vets**	**Private vets**
Crop-livestock	11.5	32.5	14.6	68.0	49.6	20.2
Pastoral	40.1	28.2	15.5	25.7	16.9	4.9
Agropastoral	30.3	31.4	18.6	36.1	30.3	4.7
Gender of HH head						
Female	24.0	24.9	12.0	47.9	37.2	11.1
Male	26.6	32.1	17.0	44.5	32.8	10.6
Herd size category						
Small (2.8)	14.8	20.0	9.0	51.1	36.0	9.7
Medium (10.5)	17.5	33.1	19.1	60.3	44.2	15.9
Large (25.7)	26.1	36.6	21.5	46.9	35.6	12.4
Very large (99.8)	45.2	33.9	15.2	23.4	19.4	5.2
Age category (average)						
1st quartile (29.9)	26.3	28.9	16.6	39.8	30.5	8.6
2nd quartile (39.4)	24.6	30.5	17.6	46.5	34.1	10.3
3rd quartile (47.0)	28.2	31.9	14.3	48.4	34.6	12.0
4th quartile (61.4)	25.0	32.5	16.0	47.5	36.1	12.4

### Effective Demand for Services

Effective demand for services was evaluated through two proxy variables, namely number of visits to service providers and health expenditures over a year. All the determinants evaluated (Table 6) significantly determined the number of visits a livestock keeper made to service providers. Highland crop-livestock farmers used the services provided more often than pastoralists (odds ratio = 2.93; *P* = 0.000), but the difference between pastoralists and agropastoralists was not significant (odds ratio = 1.025; *P* = 0.228). The average number of visits made by a crop-livestock farmer, agropastoralist and pastoralist in the year preceding the surveys were 11.1, 3.0, and 3.3, respectively. Pastoralists, however, paid significantly more per service/visit (USD 3.05) than both crop-livestock farmers (USD 1.38) and agropastoralist (USD 2.36) (Table 7).

**Table 7:** The odds of [Exp(B)] a livestock keeper from the different livestock systems, gender and age categories and service providers reporting a higher number of visits in reference to those in the reference category within each parameter.

**Parameter**	**β**	**SE**	**Sig (*P*)**	**Exp(β)**
Intercept	0.041	0.0388	0.296	1.041
System = Crop-livestock	1.074	0.0182	0.000	2.928
System = Agropastoral	0.024	0.0201	0.228	1.025
System = Pastoral	0^a^	.	.	1
Male HH head	−0.028	0.0109	0.011	0.973
Female HH head	0^a^	.	.	1
Herd size				
Medium (10.5)	−0.126	0.0106	0.000	0.882
Large (25.7)	−0.676	0.0147	0.000	0.509
Very large (99.8)	−0.034	0.0183	0.062	0.966
Small (2.8)	0^a^	.	.	1
Age of HH head (average)				
1st quartile (29.9)	−0.22	0.0128	0.000	0.803
2nd quartile (39.4)	−0.195	0.0132	0.000	0.822
3rd quartile (47.0)	−0.016	0.012	0.191	0.984
4th quartile (61.4)	0^a^	.	.	1
CAHWs	0.352	0.039	0.000	1.422
Drug shop	0.758	0.0354	0.000	2.133
Traditional healer	0.54	0.0414	0.000	1.716
Extension agent	2.482	0.032	0.000	11.971
Public vets	0.498	0.0354	0.000	1.646
Private vets	0^a^	.	.	1

a *Set to zero because this parameter is redundant*.

While the frequency of visits to the private veterinary clinics was significantly lower than to the other service providers (Table 6), the average service fee per service was reported to be significantly higher (Table 7). The average number of visits and service fees ranged from 1.8 and USD 3.77 for the private veterinarian to 19.2 and USD 0.61 to livestock extension agents. Female livestock keepers made more visits compared to males (9.2 vs. 6.5) but paid significantly less (USD 1.18 vs. 2.27). Small livestock keepers visited health centers more frequently but paid the least amount (Table 8).

**Table 8:** The odds of [Exp(B)] a livestock keeper from the different livestock systems, gender and age categories and using the different service providers expending a higher amount of money in reference to those in the reference category within each parameter.

**Parameter**	***B***	**SE**	**Sig (*P*)**	**Exp(*B*)**
Intercept	4.56	0.16	0.00	95.66
System = Crop-livestock	−0.39	0.07	0.00	0.68
System = Agropastoral	−0.17	0.05	0.00	0.84
System = Pastoral	0^a^	.	.	1.00
Male HH head	0.40	0.09	0.00	1.49
Female HH head	0^a^	.	.	1.00
Herd size				
Medium (10.5)	−0.13	0.01	0.00	0.88
Large (25.7)	−0.68	0.01	0.00	0.51
Very large (99.8)	−0.03	0.02	0.06	0.97
Small (2.8)	0^a^	.	.	1.00
HH age category (average)				
1st quartile (29.9)	−0.03	0.06	0.64	0.97
2nd quartile (39.4)	0.02	0.06	0.77	1.02
3rd quartile (47.0)	0.06	0.05	0.31	1.06
4th quartile (61.4)	0^a^	.	.	1.00
CAHWs	−1.53	0.14	0.00	0.22
Drug shop	0.12	0.06	0.06	1.12
Traditional healer	−2.10	0.31	0.00	0.12
Extension agent	−1.82	0.15	0.00	0.16
Public vets	−0.87	0.09	0.00	0.42
Private vets	0^a^	.	.	1.00

### Satisfaction With Services

Satisfaction of livestock keepers with services was evaluated based on four measures, namely availability (av), accessibility (ac), quality (qw), and timeliness (tm) of services. The average scores (out of 10) for av, ac, qw and tm were 6.1, 5.9, 6.2, and 5.7, respectively. Principal component analysis, conducted to derive a latent variable “satisfaction” from the four measures, extracted only one factor, indicating the four variables are measuring the same construct (satisfaction). Regressing the latent variable satisfaction on the four measures gave significant (*P* = 0.000) *b*-values of 0.22, 0.20, 0.13, and 0.14 for av, ac, qw, and tm, respectively, indicating strong relationships between the latent variable satisfaction and its measures.

**Figure 2:**
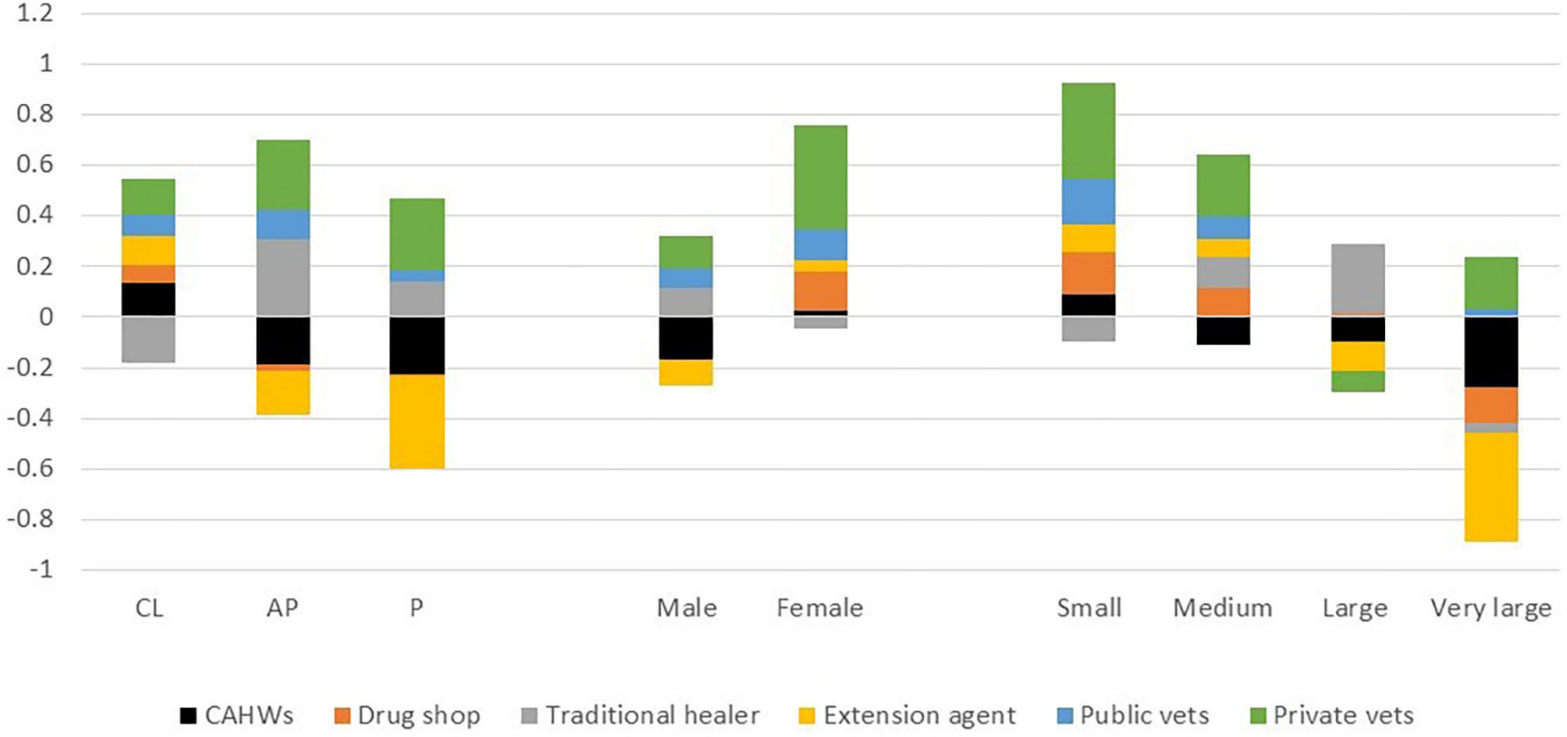
Satisfaction scores allocated by male and female respondents keeping small, medium, large and very large herds in crop-livestock (CL), agropastoral (AP), and pastoral (P) systems for services provided by different service providers in Ethiopia (scores presented are based on the first principal component factor).

Based on the first factor extracted, livestock keepers are most satisfied with the private veterinary clinics with the highest score of 0.22, followed by the public veterinary service and traditional healers with scores of 0.083 and 0.067, respectively. The private veterinarians provided the most satisfactory service across all livestock production systems and for all socio-economic groups, except for livestock keepers with very large herds (Figure 2).

## Discussion

Improved livestock productivity is largely a function of high-quality, efficient and sustainable provision as well as strong governance of veterinary services. Veterinary services, in turn, are influenced by a multitude of determinants/factors stemming from the farmers behavior toward the demanded veterinary services. The present study revealed that animal health services in the various livestock production systems of Ethiopia remain far below satisfactory standards, in line with the OIE assessment of the PVS (16) of the livestock sector (17) and animal health situation analysis of the country (5). In general, the performance of the animal health services in Ethiopia could be categorized unsatisfactory in reference to OIE's four evaluation pillars (16), including the absence of continued professional development program (CPD) and public-private partnership (PPP) for delivery of animal health services, although initiatives are underway to develop CPD program and PPP models (18).

### Veterinary Services and Service Providers

The survey findings indicated that the veterinary services provided to livestock keepers encompass disease control approaches (including modern clinical services and traditional healings) and preventive measures (vaccination), with little emphases given to parasite/pest control, disease outbreak investigation/information management, awareness/advises and training specially to the pastoral and agro-pastoral livestock rearing communities about herd health. This is in contrast to priorities reported by livestock keepers.

There is ample information disclosing critical gaps of the veterinary services in Sub-Saharan (SS) and the Greater Horn of Africa (GHA), especially with regards to the investigation, reporting, management and rapid response to livestock disease outbreaks (7, 19–21). In Ethiopia, livestock disease surveillance and reporting is not only poor but very irregular, with only 30–35% of administrative zones submitting monthly disease outbreak reports (5, 9). The situation is worse for pastoral and agro-pastoral areas (below 5%) where the sensitivity, specificity and timeliness of the reports are very low (5). Therefore, in view of the Ethiopian Livestock Master Plan there is a need to establish a robust animal health information system by improving the quantity and quality of disease outbreak and inspection reports, and conducting risk-based active surveillance on selected Transboundary animal diseases (TADs).

The present survey essentially identified major determinants governing the delivery of veterinary services, including accessibility, type of service providers, effective demand and satisfaction by livestock keepers, among others. It is widely accepted that the delivery of quality veterinary services within specific agro-ecology/production system is influenced by several factors, including farmers' perceptions toward the services, wealth status and education level of the household heads, among others (22–25).

The current study highlighted the vital role of private service providers (including CAHWs and private vets/paravets) in the veterinary service provision. Under effective training and close monitoring, CAHWs have been one of the most effective development agents to deliver house-to-house clinical services, vaccination services, control of parasites (deworming and spraying), as well as disease outbreak investigation and reporting particularly in the remote, marginal areas of the pastoral regions of Ethiopia (5, 15, 26). Even more so, their training needs to be carefully looked after to ensure that services provided are of quality and fulfill their purpose.

However, this survey clearly disclosed the little attention given to mitigating the effects of major GIT and ecto-parasites in the respective bio-geographic zones. This is in agreement other research findings in the Ethiopian highlands (23, 27–29), as well as agro-pastoral and pastoral agro-ecologies (5, 30) where the impacts of infectious and parasitic diseases on the livestock sub-sector remain high to the present date. In consequence, the national leather industry has been seriously damaged due to poor quality of skin and hide. For this reason, it is compulsory to strengthen grassroots-level animal health extension services to control/prevent the spread and deleterious effects of parasitic diseases, through the identification of areas of risk, the preparation of animal health knowledge kit, and sharing of good practices among the farming communities.

Similarly, veterinary drug shops were exposed providing unlicensed clinical services, as reported by nearly 40% of the respondents. This is an illegal act and serious violation of the existing Ethiopian regulations, which strictly prohibit private pharmacy entities to deliver clinical services whatsoever. It is not unusual to witness the private veterinary pharmacies/drug venders, in remote rural areas, engaging in drug smuggling, providing a mix of veterinary products and herbicides/pesticides, insecticides, and even medical formulations. There are increasing evidences of the misuses of drugs among the various actors including veterinary and public health, which has strongly contributed to the worsening of Anti-microbial Resistance in the field (31–33). It implies that pertinent veterinary authorities should implement strong monitoring strategies of public regulations especially at the grassroots level.

### Access to Veterinary Services

Generally, accessibility, availability and affordability of veterinary services and goods are inherent parameters which determine the quality of animal health care systems. Yet, this household survey revealed that the coverage and access of livestock keepers to veterinary services substantially varied across livestock systems, though access is relatively better in the crop-livestock systems in reference to the lowlands. This is to be expected as the available information indicates the relative concentration of the national veterinary personnel and basic infrastructure along with other logistics in the crop-livestock system mainly found in the densely populated central highlands of the country (5, 9). Moreover, livestock owners in these areas are better-off in terms of access to improved extension systems, credit/saving and other inputs services (1, 5, 27, 28). Research findings in other countries in East, West, Central and South Africa, and Asia have also revealed the strong differences in farmers' access to animal health services across different agro-ecological zones and production systems (19–22, 25, 34). These findings may also indicate that the way animal health systems are defined with sedentary livestock production systems and thus likely fail to address needs of more mobile pastoralist communities.

Indeed, the study has found that better access was reported in crop livestock and services are found to be least accessible to pastoral production systems. One of the reasons for poor access to veterinary services in pastoral areas is due to the fact that veterinary services in the pastoral areas are being delivered according to extension packages tested in sedentary production systems (i.e., crop-livestock system) and have therefore proved to be impractical and unsustainable (35). The other reason for the poor access to veterinary services in pastoral areas is due to mobility of the pastoralists, its remoteness and poor infrastructure that denies employment of professionals in arid, remote and marginal pastoral areas. Budget limitations, underdeveloped infrastructures and weak institutional arrangement are some of the problems associated with poor access to veterinary services in pastoral areas of the country.

According to this study, delivery of veterinary services in the remote and marginal lowlands of Ethiopia has been facing severe challenges in accessing affordable and reliable veterinary services (an average of only 19.3%). There are concrete reports and research findings indicating the fact that delivery of animal health services in marginalized areas have been hampered by a multitude of challenges including lack of resources by government and the low incentives for setting up private practices (7, 26, 30, 34, 36). In view of helping these communities out of chronic poverty, and realizing the national Livestock Master Plan, there is an urgent, need for commitment to enhance veterinary services in pastoral areas through accredited, nationally harmonized, and transparent community-based animal health system linked with veterinary support, involving the coordination of all agencies operating in the sector, including private service providers.

The business environment, including the highly subsidized service by the public sector, does not seem to encourage the private sector to participate, particularly in remote pastoral areas. Although the policy basis has been laid, including the “Public Private Partnership Proclamation No. 1076/2018” issued on the 22 February 2018, the “Animal Diseases Prevention and Control Proclamation No. 267/2002” and the Veterinary Services Rationalization Road Map in 2014, there has not been much progress in developing favorable legislative framework to promote participation of the private sector and the road map is yet to be ratified by the Government of Ethiopia.

### Effective Demand and Satisfaction With Services

Persistent farmers' demand for veterinary services among alternative providers is governed by accessibility, availability, quality, affordability, and timeliness of the services. The survey revealed livestock keepers (regardless of demographic attributes, animal herd size, etc.) more frequently visiting public veterinary entities than the private counterparts. This can be explained mainly in terms of the limiting national livestock policy in which, which until recently, considered veterinary services as public goods, providing little incentives to farmers to seek private services This can imply the policy favoring high subsidies or even exempting service charges which could be a driving factor to attract the community to seek public services and prevent private sector actors to enter the market. Despite considerable improvements in food security and household income over the last decade, this government scheme has highly contributed to build dependency syndrome among the community in relation to veterinary services.

Yet, there are promising government commitments to expand the private engagement in veterinary services delivery. A consultative study conducted by EVA through the EU-funded LVC/PPD project has shown incremental roles of the private sector in the veterinary domain (37), supporting the above. There are also other research findings which point the opportunities attracting farmers toward livestock rearing, including availability of reliable veterinary services, market access, extension services, etc. (5, 20, 21, 23, 27).

Supporting this push toward private sector involvement, are the findings of the multinomial regression analysis in this study, which singled out private veterinary clinics as the most satisfactory service providers for the majority of livestock keepers in all the livestock production systems, despite the highest service charges. This can be explained in relation to the frequent absence of vet supplies in most government entities as the result of budgetary constraints, negligence and recurrent turnover of veterinary personnel, and public bureaucratic issues along the supply chain. On the other hand, private veterinary service providers spend most of their time on-duties, in view of maximizing their profits and expanding their enterprises. The shortage of veterinary supplies is more severe in remote pastoral areas which results in higher cost of services as shown in the higher expenditure of pastoralists in the current study.

## Summary and Conclusions

In summary, the present study identified access to better veterinary services and types of providers, effective demand and satisfaction of the livestock keepers as the major determinants of veterinary service delivery in various livestock systems of Ethiopia. In the absence of well-documented information about these factors, this survey will undoubtedly act as the milestone for the national efforts to implement and enhance the livestock master plan in view maximizing the economic outputs from the huge livestock sub-sector. With active government policy support, livestock sector will radically transform (with moderate to high level of intensification) to respond to the increasing demands in Ethiopia. In this regard, the present study would contribute to the efforts for rationalization of veterinary service delivery. In the face of multitude opportunities, challenges and uncertainties, there is a need to expand the role of private veterinary services, with the public actor eventually capitalizing its roles mainly on regulatory and capacity building issues.

## Data Availability Statement

The data analyzed in this study is subject to the following licenses/restrictions: The data will be availed upon reasonable request and in consultation of the stakeholder institutions participating in the study. Requests to access these datasets should be directed to s.gizaw@cgiar.org.

## Author Contributions

BW and BG designed and led the surveys. MW, SG, and HA coordinated/collected the data. GG sourced funds and coordinated surveys (excluding HEARD project survey). BW initiated and designed the concept for the paper. SG analyzed the data. SG and BW drafted the paper. FA and GA drafted introduction and discussion sections. All authors read, commented, and approved the final manuscript.

## Conflict of Interest

The authors declare that the research was conducted in the absence of any commercial or financial relationships that could be construed as a potential conflict of interest.
